# Changes in women's dietary diversity before and during pregnancy in Southern Benin

**DOI:** 10.1111/mcn.12906

**Published:** 2019-12-12

**Authors:** Diane R. A. Djossinou, Mathilde Savy, Nadia Fanou‐Fogny, Edwige Landais, Manfred Accrombessi, Valérie Briand, Emmanuel Yovo, D. Joseph Hounhouigan, Agnès Gartner, Yves Martin‐Prevel

**Affiliations:** ^1^ Nutripass, Institut de Recherche pour le Développement (IRD) Université de Montpellier Montpellier France; ^2^ Faculté des Sciences Agronomiques de l'Université d'Abomey‐Calavi (FSA/UAC), Campus d'Abomey‐Calavi, Cotonou Bénin; ^3^ UMR 216‐MERIT, Institut de Recherche pour le Développement (IRD) Université Paris Descartes Paris France

**Keywords:** Benin, dietary diversity, preconception, pregnancy, nutrition

## Abstract

Dietary diversity before and during pregnancy is crucial to ensure optimal foetal health and development. We carried out a cohort study of women of reproductive age living in the Sô‐Ava and Abomey‐Calavi districts (Southern Benin) to investigate women's changes in dietary diversity and identify their determinants both before and during pregnancy. Nonpregnant women were enrolled (*n* = 1214) and followed up monthly until they became pregnant (*n* = 316), then every 3 months during pregnancy. One 24‐hr dietary recall was administered before conception and during each trimester of pregnancy. Women's dietary diversity scores (WDDS) were computed, defined as the number of food groups out of a list of 10 consumed by the women during the past 24 hr. The analysis included 234 women who had complete data. Mixed‐effects linear regression models were used to examine changes in the WDDS over the entire follow‐up, while controlling for the season, subdistrict, socio‐demographic, and economic factors. At preconception, the mean WDDS was low (4.3 ± 1.1 food groups), and the diet was mainly composed of cereals, oils, vegetables, and fish. The mean WDDS did not change during pregnancy and was equally low at all trimesters. Parity and household wealth index were positively associated with the WDDS before and during pregnancy in the multivariate analysis. Additional research is needed to better understand perceptions of food consumption among populations, and more importantly, efforts must be made to encourage women and communities in Benin to improve the diversity of their diets before and during pregnancy.

**Key Messages**

- Dietary diversity scores of reproductive age women were low in semiurban areas of Southern Benin and less than 41% of women reached the minimum dietary diversity for women.
- Women's dietary diversity scores did not change during pregnancy compared with the preconception period, with small variations in the consumption of some food groups such as eggs, dairy products, fruits, and dark green leafy vegetables.
- The absence of change in women's dietary diversity scores was mainly due to socio‐economic constraints and might be determined by dietary restrictions related to strong socio‐cultural beliefs.

## 1 INTRODUCTION

The transition from the Millennium Development Goals to the Sustainable Development Goals in 2015 placed the health and well‐being of women and adolescents at the centre of the global agenda (De‐Regil, Harding, & Roche, 2016; Mason et al., 2014). Many of the global nutrition efforts in recent years have focused on women during pregnancy and children during their first 2 years of life—the so‐called “1,000 days” period that is considered a window of opportunity to improve both maternal and children's outcomes in a sustainable manner (Mason et al., 2014; Sharma et al., 2017). Even though dietary intake and nutritional status of women during preconception are essential determinants of a healthy pregnancy as well as optimal foetal growth and development, data regarding diet in the preconception period remain scarce (Dean, Lassi, Imam, & Bhutta, 2014). Studies on the continuum before and during pregnancy are even scarcer, in particular in low‐ and middle‐income countries.

In the literature, randomised and observational studies related to pregnancy, and less frequently to the preconception period, focused primarily on women's micronutrient status and supplementation, in particular with regard to multivitamins, iron, and folic acid (Khan et al., 2011; Potdar et al., 2014; Salcedo‐Bellido et al., 2017; Sengpiel et al., 2014; Zheng et al., 2015). For example, a randomised trial in Bangladesh showed that early multimicronutrient supplementation in pregnancy reduces the occurrence of stunting in boys during months 0–54, but not in girls (Khan et al., 2011). However, little attention has been paid to women's overall diet quality during preconception and gestation, particularly concerning dietary diversity, which has been shown to be associated with greater probability of micronutrient adequacy (Martin‐Prevel et al., 2017). Poor dietary diversity during pregnancy has been documented in many contexts, particularly in low and middle‐income countries (Lee, Talegawkar, Merialdi, & Caulfield, 2013). A lack of nutritious foods, as well as low socio‐economic levels, are recognised as primary constraints in such contexts (Huybregts, Roberfroid, Kolsteren, & Van Camp, 2009). Dietary behaviours may also be responsible for changes in food consumption pattern during pregnancy. Several studies showed that beliefs about certain foods, cultural taboos, misinformation, lack of knowledge, personal aversion, and lack of appetite could affect women's diets during pregnancy (Huybregts et al., 2009; Kavle & Landry, 2018; Riang'a, Nangulu, & Broerse, 2017).

Increasing our knowledge of changes in women's dietary diversity and their determinants is necessary to design effective long‐term nutrition strategies that would optimise pregnancy and foetal outcomes. In this research, we used an original preconceptional cohort design to examine changes in women's dietary diversity from preconception to pregnancy and to investigate their environmental, social, demographic, and economic determinants in Southern Benin.

## 2 METHODS

This study was part of the Retard de croissance intra‐utérin et paludisme (RECIPAL) cohort study, which has been fully described elsewhere (Accrombessi et al., 2018). Nonpregnant women of reproductive age were recruited at a community level from Sô‐Ava and Abomey‐Calavi, two semiurban districts of Benin, and followed up with monthly until they became pregnant; these women constituted the primary cohort (preconceptional follow‐up). The subsample of women who became pregnant was then tracked monthly at the maternity clinic from early pregnancy to delivery; they constituted the secondary cohort (gestational follow‐up). The present study collected dietary intakes of women from both the primary and secondary cohorts between November 2014 and December 2017.

The RECIPAL study was approved in Benin by the ethical committees of the Institute of Applied Biomedical Sciences and the Ministry of Public Health, and in France by the French National Research Institute for Sustainable Development (IRD). The study was conducted according to the Helsinki Declaration for medical research. Before data collection, written informed consent was obtained from each participant after ensuring their understanding of the purpose, objectives, confidentiality rules, benefits, and risks of taking part in the study.

The study took place in four subdistricts in Southern Benin as follows: So‐Ava, Houedo‐Aguekon, Vekky in the district of So‐Ava, and Akassato in the district of Abomey‐Calavi. Both districts are semiurban areas, but Sô‐Ava has the distinction of being a lake area mainly occupied by natives, whereas Abomey‐Calavi is more heterogeneous in terms of population. The climate is subequatorial and characterised by a long rainy season (April–July), a short dry season (August–September), a short rainy season (September–October), and a long dry season (November–March).

Women were enrolled in the primary cohort when they met the following criteria: being 18–45 years old, married, nonpregnant, apparently healthy, not known to be sterile, using no current contraception, having no travel plans of more than 2 months during the 18 months after inclusion, willing to become pregnant, and planning to deliver in either the Sô‐Ava or Abomey‐Calavi districts. These women were visited at home every month and tested for pregnancy. Women with positive pregnancy tests were enrolled in the secondary cohort. Women who did not conceive after 1 year of follow‐up were invited to the district maternal care centre for a medical examination. In cases of genital infection, they received medical advice and were referred to a gynaecologist. Follow‐up stopped after 2 years when women did not become pregnant.

Demographic and socio‐economic characteristics of both women and their households were collected once upon inclusion in the primary cohort via a structured questionnaire. Data included household size, assets, housing type, women's ages, parity (number of children, alive or dead), type of union (polygamous/monogamous), ethnic group, education, and main activities. A multiple correspondence analysis (Sourial et al., 2010; Traissac & Martin‐Prevel, 2012) using socio‐economic data was performed to compute a wealth index and to classify households into low, middle, and high wealth levels according to tertiles.

Dietary assessments of women were conducted before conception and at each trimester of pregnancy. The minimum number of dietary assessments per woman considered in this analysis was two, and the maximum was four. One quantitative 24‐hr dietary recall (Gibson, Charrondiere, & Bell, 2017; Gibson & Ferguson, 2008) was performed through face‐to‐face interviews. Women were asked to describe all foods, drinks, and snacks consumed over the last 24 hr, including a detailed description of the recipes. Food items consumed were classified into 10 food groups according to recommended classifications (Food and Agriculture Organization [FAO] & Family Health International 360, 2016): (a) grains, white roots and tubers, and plantains (also known as starchy staples); (b) pulses (beans, peas, and lentils); (c) nuts and seeds; (d) dairy; (e) meat, poultry, and fish; (f) eggs; (g) dark green leafy vegetables; (h) other Vitamin A‐rich fruits and vegetables; (i) other fruits; and (j) other vegetables. The number of food groups consumed was summed up and dichotomised using a cut‐off at five food groups to compute the minimum dietary diversity for women (MDD‐W) indicator, which has been recently developed and validated as a proxy of micronutrient adequacy (FAO & Family Health International 360, 2016; Martin‐Prevel et al., 2017). We also used the number of food groups consumed as a continuous variable, namely the women's dietary diversity score (WDDS‐10), which ranged from 0 to 10 food groups.

Four additional food groups—red palm oil, other oils and fats, sugar and sugary drinks, and alcoholic beverages—were used for the purpose of describing women's dietary patterns. These groups were not used in the calculation of WDDS or MDD‐W.

Women's height was measured at inclusion. Women's weight was measured twice during the preconceptional follow‐up, then once a month during the gestational follow‐up. Both weight and height were measured according to World Health Organization (WHO) standard procedures (Norgan, 1988). Height was measured to the nearest millimetre with a SECA 206 gauge (Hamburg, Germany). Weight was measured with calibrated electronic scales (Tefal, France) with a precision of 100 g. Body mass index (BMI) was calculated before pregnancy and women were classified as underweight (BMI < 18.5 kg/m^2^), normal (18.5 ≤ BMI ≤ 24.9 kg/m^2^), or overweight or obese (BMI ≥ 25 kg/m^2^) based on WHO classification (WHO, 2018).

Data were collected by seven enumerators (five nurses and two nutritionists) holding at least bachelor's degrees, with experience in field data collection. They were trained over 6 days on the 24‐hr recall technique, the questionnaire and tools, and anthropometric measurements. The questionnaire was pretested by the enumerators during the training and was adjusted where needed. During data collection, the enumerators were supervised daily by an experienced nutritionist doubling as the principal investigator and supported by a team of experts in nutritional epidemiology. The supervisor checked the proper completion of the questionnaires daily as well as consistency of the answers. Data from dietary recalls were entered and cross‐checked by repeated entry using the Epidata entry 3.1 software (Lauritsen & Bruus, 2004), whereas anthropometric, socio‐economic, and demographic data were entered using ACCESS 2007.

Statistical analyses were performed using Stata 13 (College Station, TX, USA). We first described the basic characteristics of the sample from the primary cohort and examined whether women who became pregnant during the project (*n* = 316) differed from women who did not (*n* = 581). We presented mean (SD) for continuous variables and frequencies (%) for categorical variables. The main analysis was restricted to women who had one assessment at preconception and at least one assessment during pregnancy (*n* = 234). The mean WDDS, the proportion of women who consumed different food groups, and the proportion of women reaching the MDD‐W were compared over the entire follow‐up using a linear mixed model (for continuous variables) or a logistic mixed model (for categorical variables) including a random intercept (the individual) to take into account repeated measurements for the same subject. In bivariate analyses, we examined factors that were associated with women's dietary diversity before pregnancy, using the WDDS as the continuous response variable in linear regression models and using. Factors tested included subdistricts (geographical factor); women's ages, household size, parity, type of union, ethnic groups, women's and their husband's education levels (socio‐demographic factors); women's and their husbands' professional activities and wealth index of the household (economic factors); women's body mass index (nutritional factor). Variables associated with the WDDS with a level of statistical significance of 0.20 were considered for the multivariate analysis. Blocks of factors were constituted based on conceptual reasons (factors belonging to a same dimension); these blocks were successively entered in the model using a manual ascending method. The final multivariate model was used to test if the WDDS changed between the visits of follow‐up (preconception, trimester 1, 2, or 3 of pregnancy). Interaction terms factor*visit were also tested in the final model to examine whether any change in the WDDS differed according to the modality of the factors. Univariate and multivariate analyses were systematically controlled for the season because of its known effect on food availability and hence on dietary diversity. Statistical level of significance was set at *p* < .05.

### 2.1 Limitations and strengths of the study

This study has some limitations. There was only one 24‐hr recall administered at each time point; for this reason, we could not survey women's habitual dietary intake. We also focused on dietary diversity, a single dimension of diet quality, and did not take into account other dimensions or food quantities. Another limitation is the lack of a qualitative survey on the socio‐cultural component for objective measurement of attitudes, behaviours, and beliefs regarding diets before and during pregnancy. Such data would have helped us gain more insight into the trends of our results. However, this was beyond the primary focus of our study, which was to investigate whether there are changes in women's dietary diversity before and during pregnancy. Further research will focus on this purpose. Nevertheless, the cohort design of the study was a real strength and constituted a unique source of data in West Africa. As this study was part of a larger study for which biological samples were collected, we believe that the high rate of lost to follow‐up was precisely due to very strong endogenic belief and mistrust towards the research team.

## 3 RESULTS

**Figure 1:**
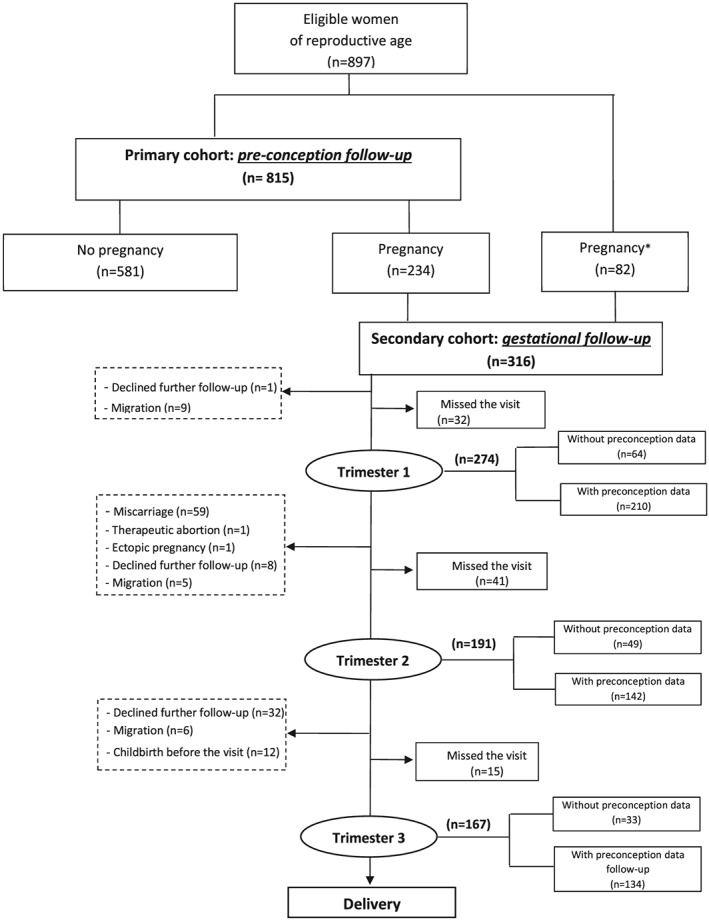
Flow diagram of participants. *Women who became pregnant after recruitment in RECIPAL and before the nutrition component started

A total of 897 women participated in the dietary intake study (Figure 1). Only 815 of those women were followed during the preconceptional period (primary cohort), because 82 women were already pregnant when the study started and hence entered the secondary cohort directly. With respect to the four time points of the dietary intake survey, the number of women varied as follows: 815 at preconception, 210 at the first trimester of pregnancy, 149 at the second trimester of pregnancy, and 134 at the third trimester of pregnancy. A total of 131 women were assessed at all four time points. From the primary cohort, 581 women did not become pregnant during the study and 234 women did.

At inclusion, women who became pregnant during follow‐up were significantly younger than those who did not (26.8 vs. 28.2 years, *p* < .001; Table 1). A higher proportion of women who became pregnant lived in monogamous households and were employed in comparison to women who did not become pregnant. There were no differences in household size, parity, ethnic group, and education between the two groups. The prevalence of overweight and obesity was high in both groups, but mostly among nonpregnant women (37% vs. 26%, *p* = .035).

**Table 1:** General characteristics of the women

Variable	Women who did not become pregnant (*n* = 581)	Women who became pregnant (*n* = 316)	*p* value
	Mean ± SD or per cent		
Subdistricts			
Vekky	34.5	28.2	<.001
Houedo‐Aguekon	12.7	—	
So‐Ava	17.5	29.1	
Akassato	35.3	42.7	
Age	28.2 ± 5.7	26.8 ± 4.8	<.001
≤24 years	26.8	29.9	<.001
25 to 29 years	31.0	43.2	
30 to 34 years	23.0	17.9	
≥35 years	19.2	9.0	
Household size	5.7 ± 3.0	5.9 ± 3.1	.814
≤ five persons	57.1	58.5	.549
> five persons	42.9	41.5	
Parity	2.8 ± 2.0	2.8 ± 2.0	.698
zero or one child	31.1	29.9	.663
two children or more	68.9	70.1	
Type of union			.002
Monogamous	61.0	73.0	
Polygamous	39.0	27.0	
Ethnic group			.158
Toffin	63.5	58.1	
Other	36.5	41.8	
Education			
No education, primary, or able to read and write	91.5	89.3	.331
Middle school or higher level	8.5	10.7	
Occupational activity			
Agriculture (self‐employed)	10.7	10.9	.011
Trade (self‐employed)	66.6	54.1	
Employee	22.9	34.6	
Body mass index (kg/m^2^)	24.1 ± 5.0	23.4 ± 4.4	.060
Underweight	7.6	7.2	.035
Normal	55.4	64.1	
Overweight or obese	37.0	28.6	

**Figure 2:**
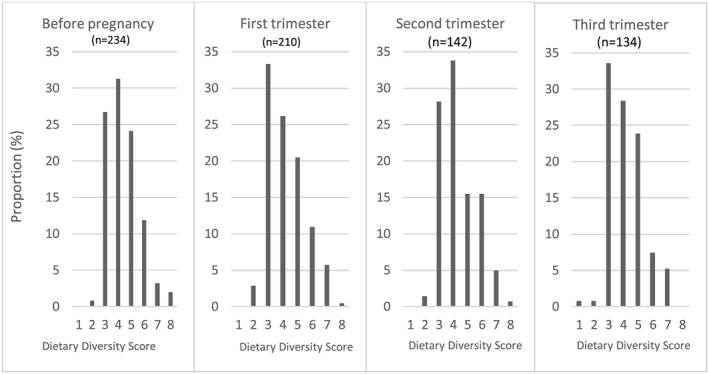
Distribution of the women's dietary diversity score before pregnancy and by trimester of pregnancy

Before pregnancy, the WDDS ranged from 2 to 8 food groups, with an average of 4.3 ± 1.1 food groups (Figure 2). The WDDS also ranged from 2 to 8 food groups at trimesters 1 and 2 of pregnancy and ranged from 1 to 7 food groups at trimester 3. The mean WDDS was 4.2 ± 1.2, 4.3 ± 1.2, and 4.1 ± 1.2 food groups in the first, second, and third trimesters of pregnancy, respectively. No statistical difference was observed in the mean WDDS according to the visit. The MDD‐W (5 food groups out of 10) was reached by 41.1% of women before conception. This proportion decreased to 37.5%, 36.9%, and 36.6% at trimesters 1, 2, and 3 of pregnancy, respectively, but none was statistically different from the preconception value.

**Figure 3:**
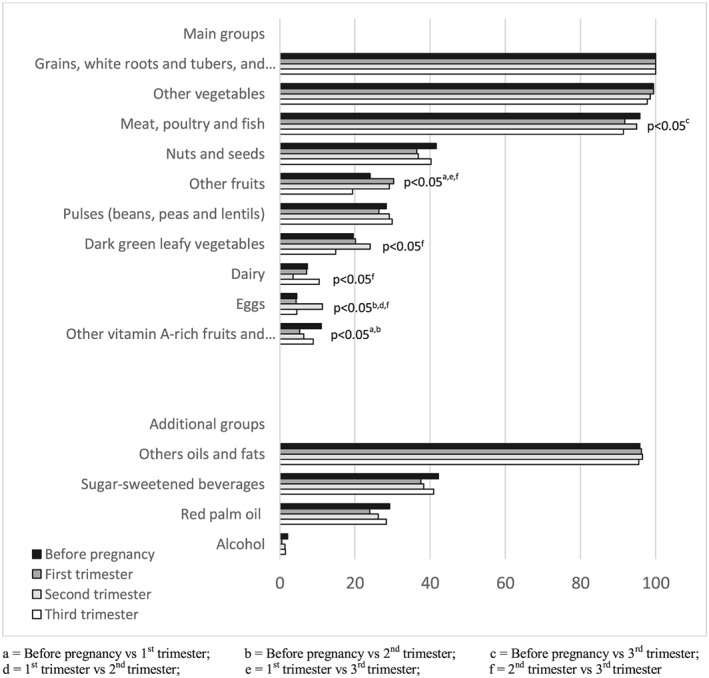
Percentages of women who consumed different food groups before and during pregnancy (*n* = 234)

Before pregnancy, as well as during pregnancy, women's diets were mainly composed of “grains, plantains, white roots, and tubers” (in particular maize, cassava, and their derivatives), “other vegetables” (mainly tomatoes, onions, and pepper), “others oils and fats” and “meat, poultry, and fish” (mainly fish and their derivatives; Figure 3). Nevertheless, the percentage of women who consumed foods from the group of meat, poultry, and fish was slightly lower during the 3rd trimester of pregnancy compared with before pregnancy (*p* = .03). Before pregnancy, the proportions of women who consumed “nuts and seeds” (mainly groundnuts, sesame seeds, and nere) and “pulses” (mainly cowpeas and Bambara nuts) were approximately 40% and 30%, respectively. These proportions did not differ statistically during pregnancy. Other fruits and “dark green leafy vegetables” were consumed by less than 30% of women before pregnancy and tended to increase at trimester 1 or 2 and to decrease back to the initial level at trimester 3 (*p* < .05). Overall, the proportion of women who consumed dairy products was very low, but it increased to 10.5% at trimester 3 (*p* = .036). Eggs were consumed by less than 5% of women, except at trimester 2 when the consumption reached 11.4% (*p* = .024). The consumption of foods from “other vitamin A‐rich fruits and vegetables” group was statistically lower during pregnancy as compared with the preconceptional period (*p* = .041). The proportion of women who consumed sugar‐sweetened beverages was not significantly different before and during pregnancy (approximately 40%).

Before conception, the mean WDDS varied across the subdistricts, with the highest level observed in Akassato and the lowest level in Vekky (Table 2). Toffin women also had lower WDDS compared with the other ethnic groups (4.2 vs. 4.5 food groups, *p* = .001), as did women with zero or one child compared with women with two or more children. The mean WDDS gradually increased with the wealth index. Women's ages, professional activities, and education levels were not associated with dietary diversity. Household size and the professional activities and education levels of the husbands were likewise not associated with the WDDS.

**Table 2:** Factors associated with the WDDS before pregnancy, controlled for the season

Variable	Preconception visit (*n* = 234)		
	*n*	Mean WDDS ± SEM	*p* value
Subdistrict			<.001
Vekky	66	3.8 ± 0.1	
Sô‐Ava	68	4.3 ± 0.1	
Akassato	100	4.6 ± 0.1	
Season			<.001
Long rainy season	18	4.5 ± 0.2	
Short dry season	130	4.1 ± 0.1	
Short rainy season	66	4.8 ± 0.1	
Long dry season	20	3.8 ± 0.2	
Age			.19
≤24 years	69	4.0 ± 0.1	
25 to 29 years	101	4.3 ± 0.1	
30 to 34 years	42	4.5 ± 0.2	
≥35 years	22	4.3 ± 0.2	
Household size			.50
≤ five persons	136	4.3 ± 0.1	
> five persons	98	4.3 ± 0.1	
Parity			.01
zero or one child	69	4.0 ± 0.1	
two or more or more	165	4.4 ± 0.1	
Type of union			.87
Monogamous	170	4.3 ± 0.1	
Polygamous	63	4.3 ± 1.1	
Ethnic group			.001
Toffin	136	4.1 ± 0.1	
Other	98	4.5 ± 0.1	
Women's education level			.20
No education, primary, or able to read and write	209	4.3 ± 0.1	
Middle school or higher level	25	4.6 ± 0.2	
Professional activity			.21
Agriculture	24	4.0 ± 0.2	
Trade	120	4.3 ± 0.1	
Employee	76	4.5 ± 0.1	
Wealth index			.01
Low	76	4.1 ± 0.1	
Medium	81	4.2 ± 0.1	
High	77	4.6 ± 0.1	
Body mass index before pregnancy (kg/m^2^)			.35
Underweight	17	4.0 ± 0.3	
Normal	149	4.3 ± 0.9	
Overweight and obese	68	4.4 ± 0.1	
Husband's education level			.18
No education, primary, or able to read and write	192	4.2 ± 0.1	
Middle school or more	42	4.5 ± 0.2	
Husband's activity			.20
Agriculture	81	4.1 ± 0.1	
Trade	21	4.5 ± 0.2	
Employee	125	4.3 ± 0.2	

Abbreviation: WDDS, women's dietary diversity score.

**Figure 4:**
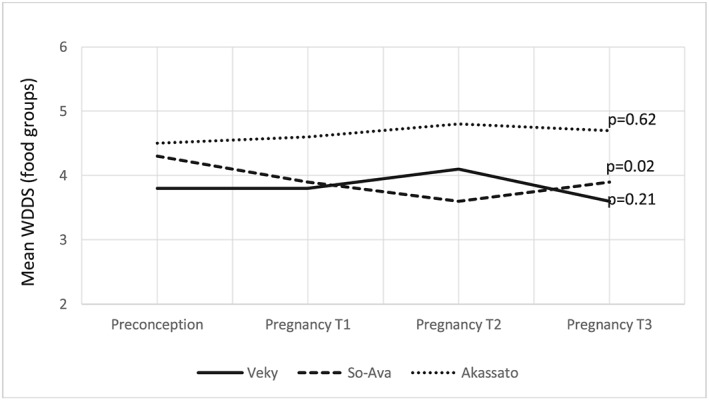
Changes in the mean women's dietary diversity score throughout follow‐up by subdistrict (Vekky, *n* = 66; So‐Ava, *n* = 68; Akassato, *n* = 100)

In the multivariate analysis adjusted for the season, subdistrict, parity, ethnic group, and wealth index, the adjusted mean WDDS ± SEM equalled 4.3 ± 0.07 food groups at preconception, 4.2 ± 0.08 at trimester 1 of pregnancy, 4.3 ± 0.09 at trimester 2, and 4.2 ± 0.1 at trimester 3, and these differences remained nonsignificant (Table 3). Only the parity and the wealth index remained positively associated with the mean WDDS in this adjusted model. The subdistrict had no effect on the mean WDDS, but the interaction term subdistrict*visit was statistically significant, suggesting that the change in the mean WDDS across the four visits was different across the subdistricts. A stratified analysis by subdistrict revealed that the mean WDDS did not statistically change over the four visits in Akassato and in Vekky, whereas it decreased during pregnancy in Sô‐Ava compared with the preconception visit, in particular at trimester 2 (Figure 4).

**Table 3:** Multivariate analysis of the change in women's dietary diversity scores from preconception to pregnancy

Variable	*n*	*β*	[95%CI]	*p* value
Subdistrict				.205
Vekky	66	ref	ref	
Sô‐Ava	68	.3181	[−0.070, 0.706]	
Akassato	100	0.3222	[−0.094, 0.738]	
Season				.582
Short rainy season	18	ref	ref	
Long dry season	130	.0967	[−0.348, 0.154]	
Long rainy season	66	.0006	[−0.256, 0.258]	
Short dry season	20	.1495	[−0.439, 0.140]	
Visit				.163
Preconception	234	ref	ref	
Pregnancy—first trimester	210	.0072	[−0.380, 0.394]	
Pregnancy—second trimester	142	.1844	[−0.251, 0.620]	
Pregnancy—third trimester	134	.3559	[−0.791, 0.079]	
Parity				.038
zero or one child	69	ref	ref	
two or more	165	.2166	[0.012, 0.421]	
Wealth index				.005
Low	76	ref	ref	
Medium	81	.2094	[−0.057, 0.475]	
High	77	.5261	[0.197, 0.855]	
Visit–subdistrict interaction				.050
Preconception at Vekky	66	ref	ref	
First trimester at Sô‐Ava	54	.4459	[−0.988, 0.096]	
First trimester at Akassato	100	.0294	[−0.457, 0.516]	
Second trimester at Sô‐Ava	31	.7684	[−1.397, −0.140]	
Second trimester at Akassato	72	.0098	[−0.555, 0.535]	
Third trimester at Sô‐Ava	39	.0334	[−0.569, 0.636]	
Third trimester at Akassato	56	.4855	[−0.080, 1.051]	

Abbreviation: CI, confidence interval.

## 4 DISCUSSION

The present study used unique cohort data to describe the dietary diversity of women of reproductive age before conception and during pregnancy in two districts of Southern Benin. Our findings showed that dietary diversity scores of women before conception were low as compared with the cut‐off of five food groups in these semiurban areas, more so in the subdistricts of Sô‐Ava and Vekky than in the subdistrict of Akassato. Barely 41% of women reached the minimum dietary diversity for women, and their diets were primarily composed of cereals, oils, vegetables, and fish. Before pregnancy, dietary diversity of women was strongly associated with the subdistrict in which they lived, the ethnic group to which they belonged, the number of children they had, and the wealth index of their household. Overall the WDDS did not vary when women became pregnant, and the scores remained low at all trimesters of pregnancy. However, in the particular setting of So‐Ava, the WDDS slightly decreased during pregnancy, in particular at trimester 2, as compared with the preconception period. The nutritional needs of pregnant women are high, and diversified diets are necessary to meet those needs.

We observed no difference in dietary diversity between the preconception and pregnancy periods, thus suggesting pregnant women do not change their diet upon learning they are pregnant. The low dietary diversity observed in preconception could therefore persist during pregnancy and put pregnant women at risk of micronutrient deficiencies with associated consequences on the baby (Hjertholm et al., 2018; Riang'a, Nangulu, & Broerse, 2017; Young et al., 2015). A study conducted in five resource‐poor settings has documented that dietary diversity was associated with micronutrient adequacy among women (Arimond et al., 2010). The link between the mother's diet and the occurrence of malformations or pathologies such as hypertension and diabetes in their children as adults has been widely demonstrated (Ramakrishnan, Grant, Goldenberg, Zongrone, & Martorell, 2012; Weber, Ayoubi, & Picone, 2015).

In the multivariate analysis, parity and household wealth index were both associated with a higher WDDS at preconception and with a positive change in the WDDS throughout follow‐up. The socioeconomic level of the household is a well‐known determinant of women's dietary diversity in African contexts (Doyle, Borrmann, Grosser, Razum, & Spallek, 2017; Huybregts et al., 2009; Krige et al., 2018; Rosen et al., 2018). Here, we showed that women with less economic constraints had better access to varied foods and were able to increase their dietary diversity during pregnancy. Women with several children were also more likely to cook every day for them and to benefit themselves at the same time. Our study did not show a significant relationship between a woman's education level and dietary diversity before or during pregnancy. This may be due to the small number of women with formal education (*n* = 25). Previous studies in some developing countries have often shown a link between the level of education of women and the quality of their diet (Huybregts et al., 2009, Mayén, Marques‐Vidal, Paccaud, Bovet, & Stringhini, 2014). The place of living also influenced women's dietary diversity. WDDS were the lowest in the subdistricts of Vekky and Sô‐Ava, probably because of a lower food availability as compared with Akassato, which is more urbanised, less isolated, and much less dependent on local food resources. However, we only observed a decrease in WDDS in Sô‐Ava during pregnancy. This may be explained by the fact that this particular area is very much rooted in tradition and beliefs. Strong socio‐cultural beliefs can lead to diet restrictions and contribute to either an absence of change or a decrease in WDDS before and during pregnancy. Studies in Burkina Faso, Kenya, Niger, and Nepal have shown that the factors that can be responsible for the change in food‐consumption patterns during pregnancy were the fear of having a large baby and beliefs held about certain foods (Christian et al., 2006; Huybregts et al., 2009; Riang'a, Nangulu, & Broerse, 2018; Rosen et al., 2018). In our study, we did observe small variations in some food group consumption. For example, the consumption of dairy products slightly increased at trimester 3 of pregnancy compared with the other visits. Extensive knowledge of the study area leads us to hypothesise that women drink a large amount of herbal teas, with or without milk, as well as other fresh dairy drinks in late pregnancy instead of eating properly, in order to reduce the size of the baby and thus avoid potential complications at delivery. Taboos about the consumption of eggs are also widespread in Africa. In some parts of Kenya, for example, the consumption of eggs during pregnancy is believed to cause obstructed labour (Riang'a, Broerse, & Nangulu, 2017). In several African countries, eating eggs may be associated with sterility (Latham, 2001). It is likely that similar beliefs about eating eggs exist in Benin. These beliefs generally concern foods or food groups that are not very frequently consumed, so the impact on dietary diversity scores was low.

Food prohibitions or restrictions during pregnancy, as well as a possible lack of awareness and knowledge about the importance of dietary diversity could be minimised by providing behaviour change communication and dietary counselling to the mothers during antenatal visits or any other communication means. However, changes in dietary behaviour are unlikely to be achieved if the whole family and community is not involved in the process (Riang'a et al., 2018). The fear of having a large baby may also be related to the risk of complications at childbirth, a situation not always well managed by the district's often understaffed and/or underequipped health centres. Improving health care quality and hence the trust of the community in the health system would also help reduce harmful beliefs about diet.

## 5 CONCLUSION

Our study used a cohort design to show that dietary diversity scores of women living in two semiurban districts of Southern Benin were low before conception and did not change during pregnancy. This lack of change was mainly due to socio‐economic constraints and possibly to negative behaviours related to socio‐cultural beliefs. Although increasing food availability and accessibility in such a context is challenging, efforts can be made to increase awareness and inform women and the whole community of the importance of a diversified diet during pregnancy.

## CONFLICTS OF INTEREST

The authors declare that they have no conflicts of interest.

## CONTRIBUTIONS

DRAD, NFF, YM‐P, AG, VB, and MA conceived and designed the study. DRAD, NFF, YM‐P, and AG designed data collection materials; DRAD, MA, and YE participated and supervised data collection; DRAD, MS, NFF, YM‐P, and AG participated in data analysis; DRAD performed the statistical analysis and had primary responsibility for the final content. MS, NFF, YM‐P, AG, EL, and DJH provided the expertise and critically reviewed the manuscript. All authors interpreted the data, revised, and approved the final manuscript.
